# Damage of the distal radial physis in young gymnasts: can three-dimensional assessment of physeal volume on MRI serve as a biomarker?

**DOI:** 10.1007/s00330-019-06247-z

**Published:** 2019-05-21

**Authors:** Rik B. J. Kraan, Laura S. Kox, Marieke A. Mens, P. Paul F. M. Kuijer, Mario Maas

**Affiliations:** 1 Amsterdam University Medical Center, Department of Radiology & Nuclear Medicine, Amsterdam Movement Sciences, University of Amsterdam, G1-229, Meibergdreef 9, 1105 AZ Amsterdam, The Netherlands; 2 Academic Center for Evidence based Sports medicine (ACES), Amsterdam, The Netherlands; 3 Amsterdam Collaboration for Health and Safety in Sports (ACHSS), International Olympic Committee (IOC) Research Center AMC/VUmc, Amsterdam, The Netherlands; 4 Coronel Institute of Occupational Health, Amsterdam Public Health Research Institute, Amsterdam University Medical Centres, University of Amsterdam, Amsterdam, The Netherlands

**Keywords:** Magnetic resonance imaging, Wrist, Growth plate, Athletic injuries, Gymnastics

## Abstract

**Objective:**

To explore the use of quantitative volume assessment to identify the presence and extent of stress-related changes of the distal radial physis in gymnasts with suspected physeal injury, asymptomatic gymnasts, and non-gymnasts.

**Methods:**

Symptomatic gymnasts with clinically suspected distal radial physeal injury, asymptomatic gymnasts, and non-gymnasts (*n* = 69) were included and matched on skeletal age and sex. Volume measurements were performed on coronal water selective cartilage MRI images by creating three-dimensional physeal reconstructions semi-automatically using active-contour segmentation based on image-intensity thresholding. Inter- and intra-rater reliability of the measurements were assessed using intra-class correlation coefficients (ICC) for absolute agreement.

**Results:**

Twenty-seven symptomatic-, 18 asymptomatic-, and 24 non-gymnasts were included with a median age of 13.9 years (interquartile range (IQR) 13.0–15.0 years). Median physeal volume was significantly increased (*p* < 0.05) in symptomatic- (971 mm^3^, IQR 787–1237 mm^3^) and asymptomatic gymnasts (951 mm^3^, IQR 871–1004 mm^3^) compared with non-gymnasts (646 mm^3^, IQR 538–795 mm^3^). Inter-rater (ICC 0.96, 95% CI 0.92–0.98) and intra-rater (ICC 0.93, 95% CI 0.85–0.97) reliability of volume measurements were excellent. Of the 10 participants with the highest physeal volumes, nine were symptomatic gymnasts.

**Conclusion:**

Increased volume of the distal radial physis can reliably be assessed and is a sign of physeal stress that can be present in both symptomatic- and asymptomatic gymnasts, but gymnasts with suspected physeal injury showed larger volume increases. Future studies should explore if volume assessment can be used to (early) identify athletes with or at risk for physeal stress injuries of the wrist.

**Key Points:**

*• The volume of the distal radial physis can be reliably assessed by creating three-dimensional physeal reconstructions.*

*• Stress-related volume increase of the distal radial physis is present in symptomatic and asymptomatic gymnasts.*

*• Gymnasts with clinically suspected physeal injury showed larger volume increases compared with asymptomatic gymnasts and may therefore be a valuable addition in the (early) diagnostic workup of physeal stress injuries.*

## Introduction

Wrist overuse injuries are prevalent among gymnasts [1]. In youth gymnasts, the distal radial physis is regularly damaged as the growth cartilage is less resistant to (repetitive) stress than surrounding structures [2, 3]. Early and accurate recognition of these physeal injuries is essential to reduce the probability of long-term consequences such as growth disturbances [4, 5].

Magnetic resonance imaging (MRI) is the imaging method of choice to evaluate the presence of physeal pathology as it is able to depict the physeal cartilage [6]. Stress-related physeal injuries are characterized by several MRI abnormalities, in particular widening of the cartilaginous part of the physis with irregularity of the borders [7, 8].

Interpretation of physeal thickness observed on MR images can be challenging as thickness is dependent on maturational status [9]. In morphological assessment of radial physeal stress injury, a comparison to the patients’ proximal physis of the first metacarpal bone (MC-1) has been proposed [10]. Maturational status influences both of these physes; however, the proximal physis of MC-1 is less subject to axial stress during gymnastics. As a consequence, using the proximal physis of MC-1 as a within-patient reference physis may be beneficial to determine if an increased distal radial physeal thickness can be attributed to maturational status or stress applied to the wrist and therefore avoid the interference of skeletal age in interpretation of distal radial physeal thickness in gymnasts [10].

We hypothesize that a systematic quantitative assessment of changes in physeal volume can be valuable for early diagnosis and for estimating the severity of stress-related physeal injuries of the wrist. Therefore, this study explores if three-dimensional MRI can serve as a biomarker for the presence and extent of stress-related changes in the volume of the distal radial physis in gymnasts with suspected physeal injury, asymptomatic gymnasts, and non-gymnasts.

## Methods

In this exploratory study, gymnasts with wrist pain suspected of having an injury of the distal radial physis, asymptomatic gymnasts, and non-gymnasts (all between 12 and 18 years old) were included between June 2015 and August 2018 after ethical approval of the study protocol by the institutional review board. Gymnasts with wrist pain were referred by a sports physician if wrist pain was present in the past 6 months and injury of the distal radial physis was suspected. Asymptomatic gymnasts were recruited via gymnastic clubs or sports physicians and included if no wrist pain had been present in the previous 6 months. All gymnasts (symptomatic and asymptomatic) had to participate in gymnastics for a period of at least 1 year. Participants for the non-gymnast group were considered eligible if they had neither participated in gymnastics in the past or present nor in wrist-loading sports more than twice a week. Asymptomatic gymnasts and non-gymnasts were matched on skeletal age and sex with symptomatic gymnasts. Exclusion criteria of all groups included a fused distal radial physis, a diagnosed growth disorder, any systematic or oncologic disease involving the musculoskeletal system, or a history of fracture, infection, or surgery of the included wrist. Each participant and his or her parents provided written informed consent before inclusion.

### Participant characteristics

Basic and clinical information of all participants was obtained using questionnaires and by performing a physical examination of both wrists. Basic information included calendar age, sex, body height, body weight, and information on the level and intensity of gymnastics- or other sports participation.

### Imaging

Skeletal age of each patient was assessed in order to evaluate the effect of skeletal age on the volume of the distal radial physis. A digital radiograph of the hand (50 kV, 3.2 mAs, focus-detector distance 1.30 m) was obtained and skeletal age was automatically determined using validated software (BoneXpert, v2.0.1.3; Visiana, www.BoneXpert.com) [11]. MRI imaging of the (symptomatic) wrist was performed using an MRI scanner with a field strength of 3.0 Tesla (Ingenia 3.0 T, Philips Healthcare) in combination with a dedicated eight-channel wrist coil. The scan protocol included a coronal T1 fast-field echo three-dimensional water selective cartilage scan (WATSc). The three-dimensional WATSc sequence provides detailed visibility of the three-layered architecture of the distal radial physis and therefore facilitates segmentation of the cartilaginous part of the distal radial physis. Parameters of the sequence were as follows: echo time 5 ms, repetition time 20 ms, slice thickness 1.5 mm, field of view 120 × 120 × 45 mm, matrix size 240 × 240, and spatial resolution 0.5 × 0.5 × 1.5 mm.

### Measurements

To quantitatively evaluate the volume of the cartilaginous part of the distal radial physis, three-dimensional physeal reconstructions were made using a semi-automatic active contour segmentation method based on image-intensity thresholding in ITK-snap (Version 3.2.0, October 23, 2014) [12]. We evaluated the volume of the proximal physis of MC-1 using the method proposed by Kox et al in the diagnostic workup of suspected pathology of the distal radial physis, comparing distal radial physeal thickness to the thickness of the proximal physis of MC-1 [10]. As MC-1 may suffer less axial loading during gymnastics, we assume that the volume of the proximal physis of MC-1 is similar in all study groups and may therefore be used as within-patient reference physis to reliably compare the volume of the distal radial physis without the interference of maturational status.

**Fig. 1:**
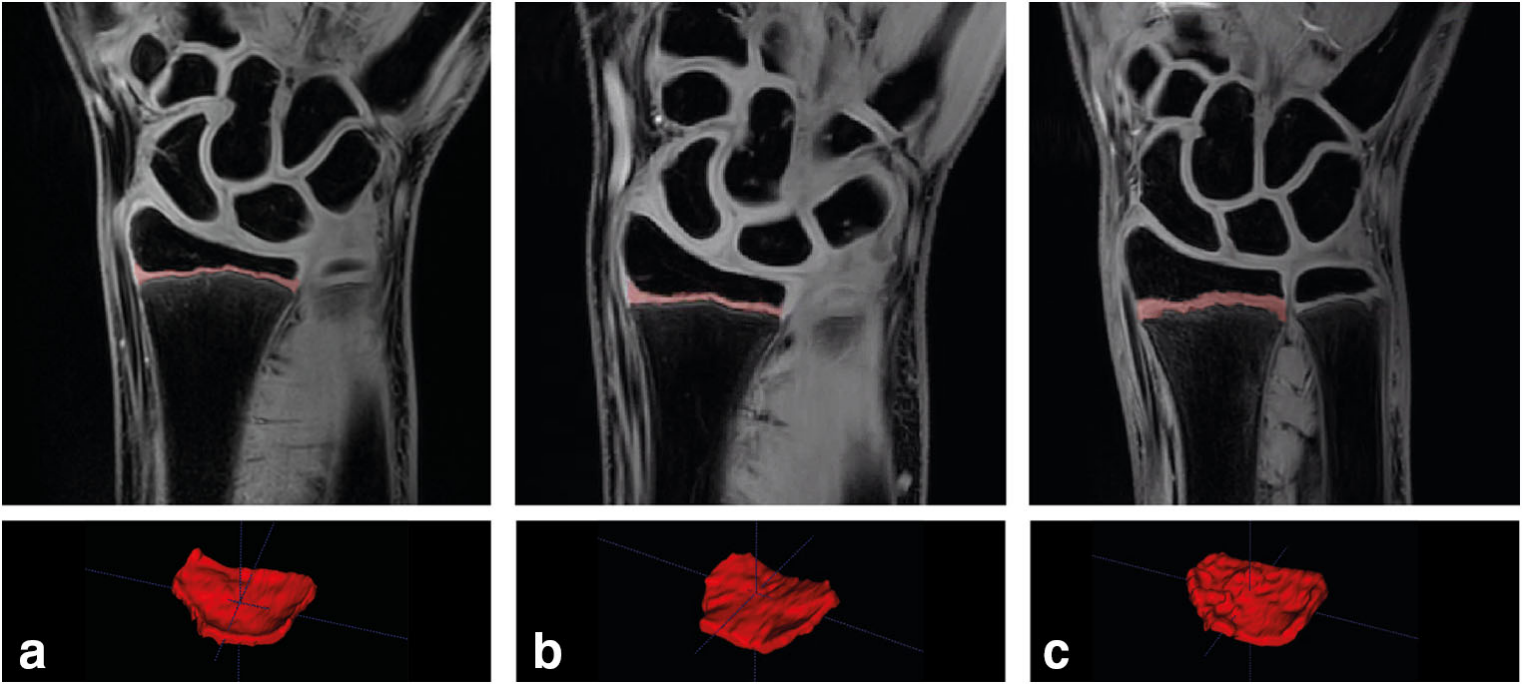
Middle slice of the wrist of a non-wrist-loading participant (**a**), an asymptomatic gymnast (**b**), and a symptomatic gymnast (**c**) on a coronal 3D WATSc image with the distal radial physis selected in red using ITK-snap and the three-dimensional reconstructed distal radial physis of the same participant

For each participant, the volumes (in mm^3^) of the two physes were calculated by one reader in ITK-snap after the three-dimensional semi-automatically segmented images (Fig. 1) were checked and adjusted manually if necessary (e.g., if areas outside the cartilaginous part of the physis were segmented). A second observer measured the volume of both physes in 30 participants (10 of each study group) to determine inter-rater reliability. The volumes of both physes of the same 30 participants were evaluated a second time by one of the readers with a minimum time interval of 2 weeks to evaluate intra-rater reliability. In addition to volume of the physes, the distances between the most medial and lateral points of the distal radial physis (in cm) were measured in RadiAnt DICOM Viewer (version 3.4.1, September 2016) to determine if changes in physeal volume were affected by possible differences in radial width.

### Data analysis

All data were entered in IBM SPSS Statistics (Version 24). Statistical tests were performed using R (RStudio, version 1.1.453). Inter- and intra-rater reliability of the volume measurements were evaluated with a two-way random intra-class correlation coefficient (ICC) for absolute agreement and the within-subject coefficient [13, 14]. Levels of agreement were defined as ICC > 0.9 = excellent, ICC 0.75–0.9 = good, ICC 0.5–0.75 = moderate, and ICC < 0.5 = poor [15]. Differences between study groups were evaluated using one-way analysis of variance (ANOVA). The Tukey or Games-Howell post hoc test was used to identify varying study groups if the results of the ANOVA were significant (*p* < 0.05). For data without a Gaussian distribution, the Kruskal-Wallis test by ranks was used with the Dunn-Bonferroni post hoc testing. To evaluate and visualize the relation between the volume of the distal radial physis and skeletal age, a scatterplot was created.

## Results

### Participants

Sixty-nine participants were included between June 2015 and August 2018 of which 33 (48%) were female. Three participants were excluded for analysis of the distal radial physeal volume as they had a fused distal radial physis on MRI (defined as a distal radial physeal volume of less than 100 mm^3^). The included study population for analysis consisted of 27 symptomatic gymnasts with suspected distal radial physeal injury, 16 asymptomatic gymnasts, and 23 non-gymnasts. No significant differences were found in participant characteristics between the groups (Table 1). Median calendar age was 14.5 years in symptomatic gymnasts, 14.0 years in asymptomatic gymnasts, and 13.3 years in non-gymnasts. Median skeletal age was 13.1 years, 11.9 years, and 13.3 years in symptomatic gymnasts, asymptomatic gymnasts, and non-gymnasts, respectively. Median age at which gymnasts started gymnastics training was 6.0 years in both symptomatic and asymptomatic gymnasts. Symptomatic gymnasts had a median training intensity of 23 h per week, and asymptomatic gymnasts trained a median 16 h per week. A full overview of participant characteristics is illustrated in Table 1.

**Table 1:** Participant characteristics, demonstrated as median (interquartile range)

	Symptomatic gymnasts	Asymptomatic gymnasts	Non-gymnasts	*p* value
	*N*			
	27	16	23	
Sex—female (%)	12 (44%)	8 (50%)	11 (48%)	n.s.
Height (cm)	158 (151–165)	158 (152–165)	165 (156–170)	n.s.
Weight (kg)	47.0 (40.5–54.0)	45.0 (41.8–49.0)	48.0 (43.0–62.0)	n.s.
BMI (kg/m^2^)	18.4 (17.0–19.5)	18.0 (17.5–18.5)	18.0 (17.0–20.6)	n.s.
Skeletal age (years)	13.1 (12.5–13.6)	11.9 (11.3–13.0)	13.3 (12.4–13.9)	n.s.
Calendar age (years)	14.5 (13.4–15.1)	14.0 (13.5–14.6)	13.3 (12.6–14.0)	n.s.
Age at start of gymnastics training (years)	6.0 (5.0–6.5)	6.0 (5.0–6.3)	NA	n.s.
Gymnastics training h/week	22.5 (12.5–28.0)	16.3 (12.1–31.6)	NA	n.s.
Gymnastics experience (years)	9.1 (7.6–10.1)	9.0 (7.1–9.3)	NA	n.s.
Gymnastics level—elite (%)	26 (96%)	14 (88%)	NA	n.s.

*NA*, not applicable; *n.s*, not significant; (*p* value > 0.05)

### Inter- and intra-rater reliability

Inter-rater reliability for volume measurements of the three-dimensional reconstructed distal radial physes was excellent with an ICC of 0.96 (95% CI 0.92–0.98) and CV of 9.1%. Intra-rater reliability for volume measurements of the distal radial physis was excellent as well with an ICC of 0.93 (95% CI 0.85–0.97) and CV of 14.6%. Inter- and intra-rater reliability of volume measurements of the proximal physis of MC-1 were both excellent with ICCs of 0.97 (95% CI 0.94–0.99) and 0.98 (95% CI 0.95–0.99) and CVs of 9.9% and 8.0%, respectively.

### Volume distal radial physis

**Fig. 2:**
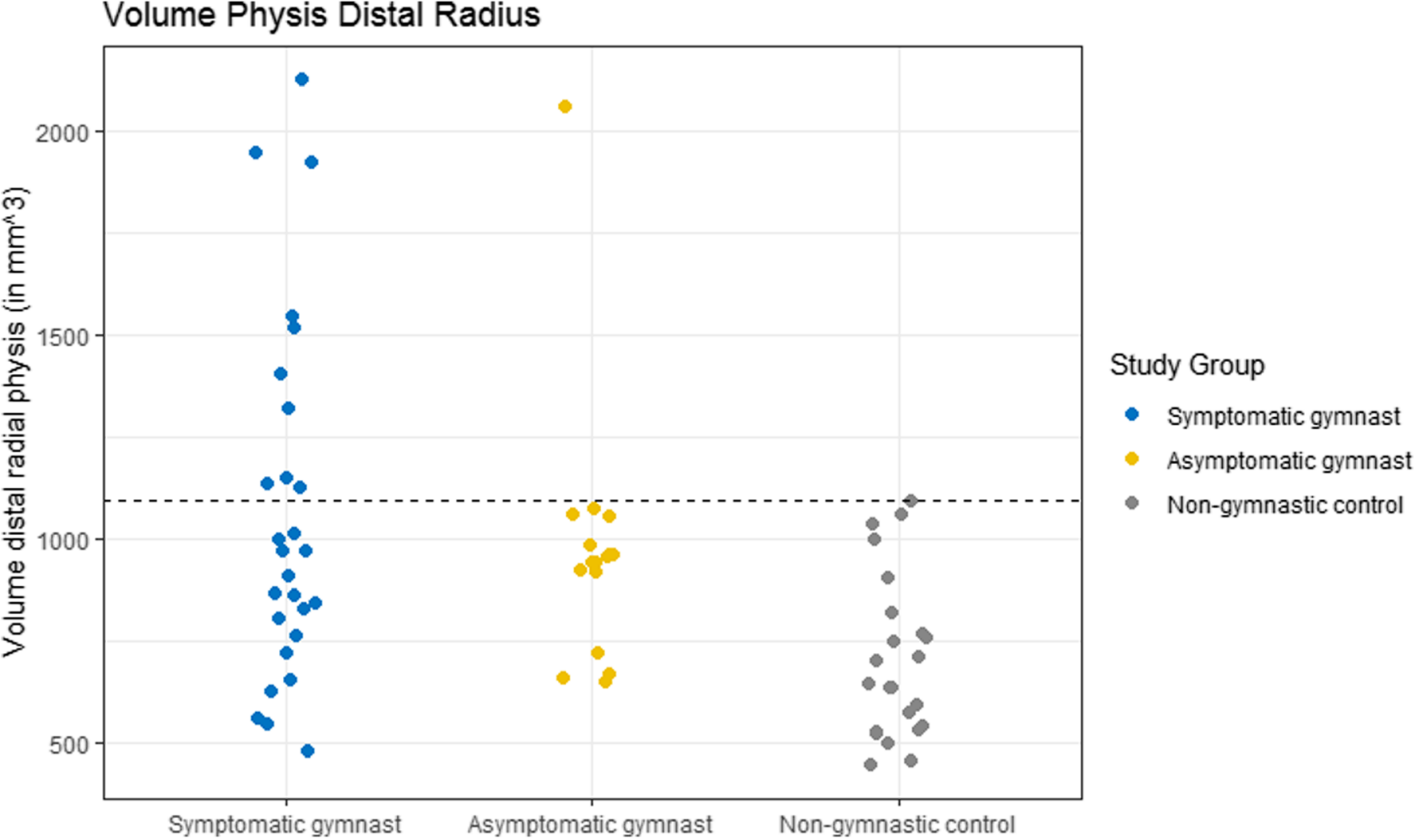
Scatterplot of the distal radial physeal volumes of all participants in the three study groups. The dashed line represents the maximum distal radial physeal volume in the non-gymnast group (1093 mm^3^)

**Fig. 3:**
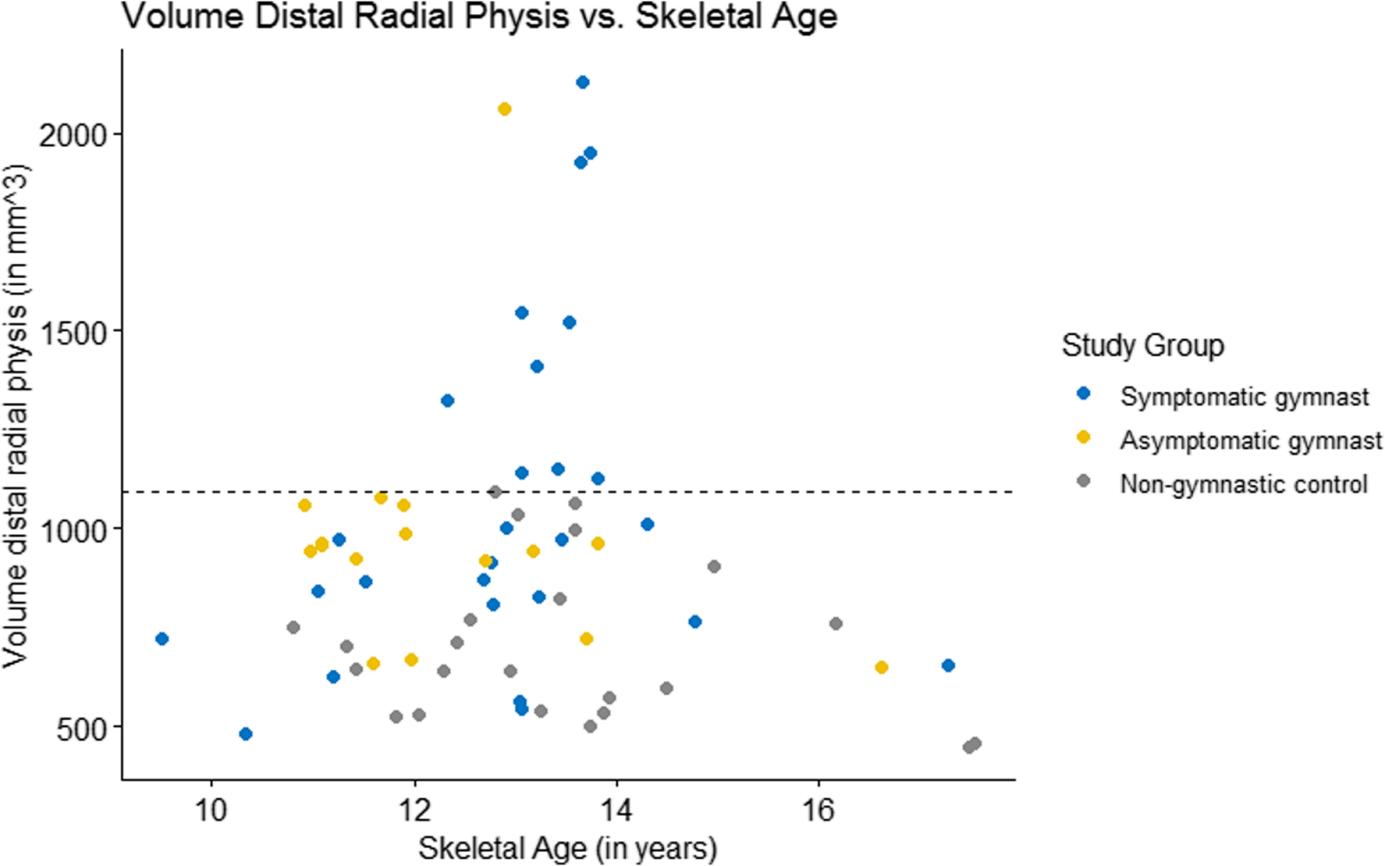
Scatterplot that demonstrates the relationship between volumes of the distal radial physis and skeletal age in the three groups

Median volume of the distal radial physis differed significantly between groups (*p* < 0.01) and was 971 mm^3^ (IQR 787–1237 mm^3^) in symptomatic gymnasts, 951 mm^3^ (IQR 871–1004 mm^3^) in asymptomatic gymnasts, and 646 mm^3^ (IQR 538–795 mm^3^) in non-gymnasts controls (Table 2). Pair-wise comparison with adjustment for multiple testing demonstrated a significantly smaller physeal volume of the distal radial physis in non-gymnasts compared with both symptomatic (*p* < 0.01) and asymptomatic gymnasts (*p* < 0.01). Figure 2 illustrates the distribution of physeal volumes in the study groups. The largest distal radial physeal volume of participants in the non-gymnasts controls was 1093 mm^3^. In 11 gymnasts, the volume of the distal radial physis exceeded the largest physeal volume of non-gymnasts (1093 mm^3^). These 11 gymnasts included 10 (91%) gymnasts with wrist pain suspected of having an injury of the distal radial physis and one asymptomatic gymnast and all had a skeletal age between 12 and 14 years (Fig. 3).

**Table 2:** Physeal measurements, demonstrated as median (interquartile range)

	Symptomatic gymnasts	Asymptomatic gymnasts	Non-gymnasts
Volume distal radial physis in mm^3^*^‡^	971 (787–1237)	951 (871–1004)	646 (538–795)
Volume proximal physis of MC-1 in mm^3^*	189 (137–212)	156 (136–194)	119 (60–154)
Medial-lateral distance distal radial physis in cm	2.84 (2.61–2.96)	2.72 (2.66–2.85)	2.73 (2.63–2.82)

*Significant difference (*p* < 0.05) between symptomatic gymnast and non-gymnast groups

^‡^Significant difference (*p* < 0.05) between asymptomatic gymnast and non-gymnast groups

### Medial to lateral width

The groups displayed no differences in medial-lateral distance of the distal radial physis (*p* = 0.59). Median distance was 2.84 cm (IQR 2.61–2.96 cm), 2.72 cm (IQR 2.66–2.85 cm), and 2.73 cm (IQR 2.63–2.82 cm) in symptomatic gymnasts, asymptomatic gymnasts, and non-gymnasts controls, respectively.

### Volume proximal physis of MC-1

Median volume of the proximal physis of MC-1 was significantly different between symptomatic gymnasts and non-gymnasts (*p* < 0.01). The volumes were 189 mm^3^ (IQR 137–212 mm^3^) in symptomatic gymnasts, 156 mm^3^ (IQR 136–194 mm^3^) in asymptomatic gymnasts, and 119 mm^3^ (IQR 60–154 mm^3^) in non-gymnasts.

## Discussion

This study illustrates that the volume of the distal radial physis is increased in gymnasts regardless of symptoms. The largest increases in volume were observed in gymnasts with clinical distal radial physeal injury, especially in participants aged 12 to 14 years. The volume of the proximal physis of MC-1 was significantly increased in symptomatic gymnasts compared with non-gymnasts.

## Interpretation of findings

In healthy individuals, physeal thickness is primarily determined by maturational status. The physis becomes thinner as maturation approaches and will eventually disappear [9, 16]. As a consequence, the assessment of physeal thickness can be challenging since delayed maturation can be easily misinterpreted as stress-related physeal volume increase.

Gymnasts included in this study demonstrated a high median number of training hours per week, namely 23 h (symptomatic-) and 16 h (asymptomatic gymnasts) besides daily school activities. This weekly training duration is higher compared with the mean amount of training hours per week in other young elite athletes, for example football players (11 h), hockey players (6 h), and speed skaters (12–17 h) [17]. Female gymnasts participating in high-intensity training programs are at risk for delayed maturation [18] and the possible influence of maturational status on physeal volume measurements should therefore be taken into account. We aimed to minimize this potential bias by matching participants based on skeletal age. The demonstrated differences in physeal volumes are therefore more likely caused by physeal stress than by variances in maturational status.

### Distal radial physis

On water-sensitive MRI sequences, a healthy physis appears as a multilayered structure, including a hyper-intense layer of physeal cartilage cells, a hypo-intense zone of provisional calcification, and a hyper-intense layer of newly formed and highly vascular metaphyseal spongiosa [9]. Stress-related physeal widening is attributed to the accumulation of chondrocytes in the physeal cartilage due to the absent or delayed endochondral calcification and can thus be seen as widening of the layer of physeal cartilage cells on MRI. The accumulation of chondrocytes is caused by damage to the metaphyseal vascularization which normally induces the endochondral calcification process in healthy growing children [19–21].

In this study, stress-related changes in distal radial physeal thickness occur in both symptomatic and asymptomatic gymnasts, but gymnasts with clinically suspected distal radial physeal injury demonstrated larger volume increases. We consider the changes in physeal thickness in asymptomatic gymnasts to be an early sign of physeal stress which may progress into more severe physeal widening if stress continues to be applied to the physis for a longer period of time. Jaramillo et al demonstrated that physeal thickness of the tibia in rabbits normalized weeks after inducing damage to the metaphyseal blood vessels of the tibia [19]. This suggests that sufficient rest is essential to reverse stress-related physeal widening and to prevent the development of physeal pathology in asymptomatic gymnasts.

### Age

Young gymnasts are at risk for wrist pain, especially between the age of 10 and 14 years [1, 22]. During these years, the immature skeletal system grows quickly in the pubertal growth spurt [23, 24]. In periods of rapid growth, the physis is extra vulnerable for stress-related injuries [25]. This could explain the fact that in our study, all gymnasts with a volume of the distal radial physis exceeding the largest volume in the non-gymnast group were between 12 and 14 years old.

### Proximal physis of MC-1

We hypothesized that volumes of proximal MC-1 physes would be similar in all study groups as differences in maturational status were absent and therefore we originally planned to use the proposed comparison between the physes of the distal radius and MC-1 as proposed by Kox et al [10]. However, the results illustrated that a significant difference in proximal MC-1 physeal volume was present between symptomatic gymnasts and non-gymnasts and a slight (non-significant) increase in the volume was present in asymptomatic gymnasts as well. Therefore, proximal MC-1 physeal volumes were not used in the analysis and interpretation of distal radial physeal thickness.

Using the proximal physis of MC-1 as a morphological reference for the thickness of the distal radial physis in gymnasts might still be useful in the diagnostic workup of physeal injuries; however, based on these quantitative findings, cautious interpretation and further validation are necessary.

## Strengths and limitations

The excellent inter- and intra-rater agreements indicate that the proposed method is reliable and that the obtained results can confidently be used for analysis. The strategy to match participants in the three study groups on skeletal age in combination with the absence of significant differences in the amount of training hours between the study groups reduces the likelihood of changes in physeal volume as a result of other factors, such as maturity status or the quantity of stress applied on the physis. In addition, the high training volume in both symptomatic and asymptomatic gymnasts ensures optimal evaluation of the effect of gymnastics on physeal volume.

The major limitation in our study is that participants in the study group with wrist pain were selected by a sports physician if an injury of the distal radial physis was suspected based on clinical findings. Consequently, bias could be introduced as in some participants suspected of having physeal injury symptoms might be caused by other pathologies. Some participants demonstrated other MRI abnormalities, for example dorsal wrist impingement. In these cases, the distal radial physis of these (symptomatic) participants could have a normal volume. However, possibly, these participants had an overuse injury of the distal radial physis simultaneously, and as no consensus exists on MRI features that confirm the presence of physeal pathology, the use of clinical symptoms as inclusion criteria was, in our opinion, the most suitable option. Furthermore, if present, this potential bias did not substantially influence the results as symptomatic gymnasts demonstrated a significant increase in distal radial physeal volume. In conclusion, the number of participants was relatively small as the study was designed as a pilot study. Despite this, significant differences were found, indicating a large effect size.

## Clinical implications

The method we used for objective and quantitative assessment of the physeal showed excellent reliability and can therefore be valuable for physeal thickness assessment in clinical practice. It is performed using an off-the-shelf MRI sequence and widely available software which facilitates the implementation. However, in cases with severe pathology with physeal irregularity, manual adjustment of the reconstructed physis can be time-consuming.

For clinical practice and for interpretation of diagnostic images in athletes with suspected physeal injuries, it is essential to realize that increased distal radial physeal volume is present in asymptomatic gymnasts as well. Possibly, these increased volumes reflect early (subclinical) physeal damage as a result of wrist-loading during gymnastics. Further studies should explore if volume changes in asymptomatic gymnasts indicate an increased risk for obtaining symptomatic physeal stress injuries, for example by prospectively monitoring distal radial physeal volume in young gymnasts over time and exploring if volume changes precede symptoms. Subsequently, the development of these injuries might be prevented by detecting gymnasts who are at risk and reducing their amount of wrist-loading activities.

Several gymnasts with clinically suspected injury of the distal radial physis demonstrated a physeal volume that exceeded the largest observed volume in non-gymnasts. We consider that the combination of large volume increase and clinical symptoms indicates the presence of physeal pathology. Therefore, the assessment of physeal volume in gymnasts suspected of physeal pathology can be a valuable addition to the current clinical diagnostic workup [10]. In addition, further studies should explore the value of physeal volume assessment in the follow-up of (conservative) therapy effects and for guiding return-to-play decision-making.

## Conclusion

The volume of the distal radial physis can be assessed reliably. Stress-related volume increase of the distal radial physis is present in gymnasts regardless of symptoms, but gymnasts with clinically suspected physeal injury showed larger volume increases. Future studies should explore if volume assessment of the distal radial physis can be used to (early) identify athletes with or at risk for physeal stress injuries of the wrist.

## Abbreviations

CV: Coefficient of variation
ICC: Intra-class correlation coefficient
IQR: Interquartile range
MC-1: First metacarpal bone
